# 酸枣仁种皮和种仁化学成分的定性及半定量分析

**DOI:** 10.3724/SP.J.1123.2023.09015

**Published:** 2024-03-08

**Authors:** Yuhao SHI, Yi NAN, Wei ZHENG, Lan YAO, Haizhen LIANG, Xiaojuan CHEN, Juan SONG, Jie ZHANG, Dexian JIA, Qian WANG, Baiping MA

**Affiliations:** 1.天津中医药大学, 天津 301617; 1. Tianjin University of Traditional Chinese Medicine, Tianjin 301617, China; 2.军事科学院军事医学研究院辐射医学研究所, 北京 100850; 2. Institute of Radiation Medicine, Academy of Military Medical Sciences, Academy of Military Sciences, Beijing 100850, China; 3.北京中医药大学, 北京 100029; 3. Beijing University of Chinese Medicine, Beijing 100029, China

**Keywords:** 超高效液相色谱, 电雾式检测器, 质谱, 差异成分, 酸枣仁, 种皮, 种仁, 半定量分析, ultra performance liquid chromatography (UPLC), charged aerosol detector (CAD), mass spectrometry (MS), difference markers, Ziziphi Spinosae Semen, seed coat, seed kernel, semiquantitative analysis

## Abstract

酸枣仁为鼠李科植物酸枣*Ziziphus jujuba* Mill. var. *spinosa* (Bunge) Hu ex H. F. Chou的干燥成熟种子,其分为种皮、种仁两个部位,比较研究酸枣仁不同部位化学成分组成及相对含量可为合理开发利用中药酸枣仁资源提供理论依据。基于超高效液相色谱-四极杆飞行时间质谱(UPLC-Q-TOF/MS)技术,从种皮、种仁中共鉴定出57个化学成分。结合主成分分析(PCA)和正交偏最小二乘法判别分析(OPLS-DA)对两者进行差异成分研究,以变量投影重要度(VIP)值> 5为标准,筛选了差异成分17个,其中白桦脂酸、桦木酮酸、麦珠子酸和酸枣仁皂苷Ⅰ主要存在于种皮部位,斯皮诺素、酸枣仁皂苷A和6‴-阿魏酰斯皮诺素等13个化合物主要存在于种仁部位。通过超高效液相色谱-电雾式检测器(UPLC-CAD)结合反梯度补偿技术,建立半定量液相色谱指纹图谱,考察了6个不同结构类型的代表成分的响应一致性,其不同浓度下平均响应因子间的RSD值为7.04%,各化合物响应一致性良好,可用于酸枣仁的半定量表征分析;结果表明:种皮部位主要成分为白桦脂酸和油酸,其中白桦脂酸的含量约是种仁的7倍;种仁部位主要成分为斯皮诺素、酸枣仁皂苷A、亚油酸、白桦脂酸和油酸,其中斯皮诺素、酸枣仁皂苷A的含量分别是种皮的18倍和24倍。综上,本研究阐明了酸枣仁种皮、种仁的化学成分差异,明确了酸枣仁两个部位中各自的主要成分及其相对含量,为酸枣仁不同部位合理开发和利用奠定了基础。

## Abstract (en)

Ziziphi Spinosae Semen refers to the dried seed of *Ziziphus jujuba* Mill. var. *spinosa* (Bunge) Hu ex H. F. Chou. The seed is composed of a reddish brown coat and a yellow kernel. A comparative study was conducted to investigate differences in the chemical composition and their relative contents between the seed coat and kernel of Ziziphi Spinosae Semen. First, the chemical compounds found in the seed coat and kernel were characterized and identified using ultra performance liquid chromatography-quadrupole time-of-flight mass spectrometry (UPLC-Q-TOF/MS). The analytical results tentatively identified 57 chemical compounds based on reference-compound comparison, literature retrieval, and chemical-database (e. g., MassBank) searches; these compounds included 14 triterpenes, 23 flavonoids, 7 alkaloids, 6 carboxylic acids, and 7 other types of compounds. The mass error of the identified compounds was within the mass deviation range of 5×10^-6^ (5 ppm). Next, two methods of multivariate statistical analysis, namely, principal component analysis (PCA) and orthogonal partial least squares-discriminant analysis (OPLS-DA), were used to compare the differential compounds between the two seed parts. A total of 17 differential compounds were screened out via OPLS-DA based on a variable importance in projection (VIP) value of >5. The results revealed that betulinic acid, betulonic acid, alphitolic acid, and jujuboside Ⅰ mainly existed in the seed coat whereas the 13 other compounds, such as spinosin, jujuboside A, and 6‴-feruloylspinosin, mainly existed in the seed kernel. Therefore, these 17 differential compounds can be used to distinguish between the two seed parts. Finally, a semiquantitative method was established using UPLC and a charged aerosol detector (CAD) with inverse gradient compensation in the mobile phase. Six representative compounds with different types were selected to examine the CAD response consistency: magnoflorine (alkaloid), spinosin (flavone), 6‴-feruloylspinosin (flavone), jujuboside A (triterpenoid saponin), jujuboside B (triterpenoid saponin), and betulinic acid (triterpenoid acid). The results showed that the relative standard deviation (RSD) of the average response factors at different levels of these six compounds was 7.04% and that their response intensities were similar. Moreover, each compound in the fingerprint demonstrated good response consistency, and the peak areas obtained directly reflected the contents of each compound. Based on the semiquantitative fingerprints obtained, betulinic acid and oleic acid were considered the main components of the seed coat. The betulinic acid content in the seed coat was approximately 7 times higher than that in the seed kernel. Spinosin, jujuboside A, linoleic acid, betulinic acid, and oleic acid were the main components of the seed kernel. The spinosin content in the seed kernel was 18 times higher than that in the seed coat. In addition, the jujuboside A content in the seed kernel was 24 times higher than that in the seed coat. The proposed method can accurately determine the main components and compare the relative contents of these components in different seed parts. In summary, this study identified the differences in chemical components between the seed coat and kernel of Ziziphi Spinosae Semen and clarified the main components and their relative contents in these parts. The findings can not only provide a basis for the identification of chemical compounds and quality research on different parts of Ziziphi Spinosae Semen but also promote the development and utilization of this traditional Chinese medicine.

中药酸枣仁为鼠李科植物酸枣*Ziziphus jujuba* Mill. var. *spinosa* (Bunge) Hu ex H. F. Chou的干燥成熟种子,其始载于《神农本草经》,被列为上品,是一味养心安神的中药。根据2020年版《中国药典》记载,酸枣仁具有养心补肝、宁心安神、敛汗、生津的功效,用于虚烦不眠、惊悸多梦、体虚多汗、津伤口渴^[1]^。酸枣仁主产于河北、山西、陕西、辽宁等地,主要化学成分包括三萜、黄酮、生物碱、多糖等^[2⇓-4]^,具有镇静催眠^[5,6]^、抗抑郁^[7]^、抗焦虑^[8]^、抗心律失常^[9]^、抗肿瘤^[10]^等药理活性。

近年来,液相色谱-质谱联用技术已广泛应用于中药成分研究,例如黄晓欣等^[11]^利用超高效液相色谱-线性离子阱/静电场轨道阱质谱技术对不同产地酸枣仁进行差异分析,李泽等^[12]^利用超高效液相色谱-四极杆-静电场轨道阱质谱与多元统计分析相结合的方法,筛选出生、炒酸枣仁潜在的差异化学成分。目前王健等^[13]^应用薄层扫描法测定了酸枣仁的种皮、胚乳和子叶中酸枣仁皂苷A(jujuboside A)和酸枣仁皂苷B(jujuboside B)的含量,但对酸枣仁种皮、种仁部位成分的系统研究较少。有研究报道酸枣仁种皮和种仁抗氧化活性存在差异^[14]^,这种差异可能来源于两者间的化学成分差异。同时从酸枣仁使用角度来看,其方式有研磨成粉冲服、煎煮等,在粉末冲服食用过程中,存在红褐色种皮涩口的情况,若剥去种皮食用,口感则明显改善。因此需要明确酸枣仁不同部位化学成分的组成与含量,为后续研究酸枣仁不同部位镇静催眠、抗氧化等药理活性奠定理论基础,也为酸枣仁的使用提供科学依据。

用于酸枣仁质量控制的液相色谱法多采用蒸发光散射检测器(ELSD)测定酸枣仁中酸枣仁皂苷A、斯皮诺素(spinosin)、酸枣仁皂苷B、6‴-阿魏酰斯皮诺素(6‴-feruloylspinosin)^[15]^,但ELSD存在一定的局限性。电雾式检测器(charged aerosol detector, CAD)是一种质量型通用检测器,其特点是能检测非挥发性和半挥发性物质,响应值不依赖于物质的结构性质,比ELSD有更高的灵敏度和精密度,对不同结构类型化合物有相对均匀的响应^[16]^。CAD雾化效率依赖于挥发性流动相的组成^[17]^,反梯度补偿法可以为CAD提供恒定的流动相组成,以保持均匀响应。郑伟等^[18]^建立了超高效液相色谱-CAD结合反梯度补偿的半定量分析方法,对不同采收时间的山楂叶进行成分分析,结果表明,山楂叶成分的半定量分析结果与绝对定量结果一致,证明该方法准确度较高。因此,结合UPLC-CAD和反梯度补偿技术的分析方法可用于建立酸枣仁的半定量指纹图谱,并可用于分析酸枣仁种皮、种仁中主要化学成分及相对含量的差异。

本实验采用10批酸枣仁样品,利用超高效液相色谱-四极杆飞行时间质谱(UPLC-Q-TOF/MS)对酸枣仁不同部位的化学成分进行定性分析,同时结合主成分分析(principal component analysis, PCA)和正交偏最小二乘法判别分析(orthogonal partial least squares-discriminant analysis, OPLS-DA)对酸枣仁不同部位进行差异性分析,筛选差异成分。同时建立UPLC-CAD半定量分析方法,明确不同部位的主要成分,阐明不同部位代表成分的含量差异,为酸枣仁不同部位化学成分的快速鉴定提供参考,也为研究不同部位酸枣仁药效差异及质量控制提供理论依据。该方法将有利于酸枣仁的有效资源开发和利用。

## 1 实验部分

### 1.1 仪器、试剂与材料

Waters ACQUITY I-Class超高效液相色谱系统、VION-IMS-Q-TOF质谱系统、UNIFI 1.9.4软件(美国Water公司); Vanquish Flex UHPLC液相色谱仪及CAD(美国Thermo Fisher公司); BP211D十万分之一天平(德国Sartorius公司); KQ-600DE数控超声波清洗器(昆山市超声仪器有限公司)

木兰花碱(magnoflorine,批号:DSTDM000401,纯度98.7%)、斯皮诺素(批号:DSTDS006002,纯度98.0%)、6‴-阿魏酰斯皮诺素(批号:DST201019-013,纯度99.3%)、酸枣仁皂苷A(批号:DST221102-058,纯度99.5%)、酸枣仁皂苷B(批号:DST220608-059,纯度98.5%)、白桦脂酸(betulinic acid,批号:DST200626-026,纯度98.2%)均购于成都德思特有限公司。乙腈、甲酸(质谱级,纯度≥99%,美国Thermo Fisher公司),蒸馏水(广州屈臣氏有限公司);无水乙醇(分析纯,国药集团化学试剂有限公司)。样品共10批,S1~S10分别来自河北(4个)、陕西(2个)、山东(2个)、山西(1个)、内蒙地区(1个),样品批号依次为XT2021070715、XT2021082501、CXT20210825、TZJ20211224、SX320211010、SX320220108、SD20210729、SD20211010、SX20220108、NM20220108, 其中S3~S4为炒品,其他批次为生品,经中国医学科学院药用植物研究所郭宝林研究员鉴定为鼠李科植物酸枣*Z. jujuba* Mill. var. *spinosa* (Bunge) Hu ex H. F. Chou的干燥成熟种子。

### 1.2 溶液制备

对照品溶液:分别精密称定对照品木兰花碱、斯皮诺素、6‴-阿魏酰斯皮诺素、酸枣仁皂苷A、酸枣仁皂苷B和白桦脂酸适量,置于10 mL容量瓶中,加入甲醇定容制成质量浓度依次为1005、1015、1014、1025、1005、1010 μg/mL的储备液;各对照品储备液分别精密量取2.5 mL,置于25 mL容量瓶中,加入甲醇定容制成质量浓度约为100 μg/mL的混合对照品溶液。再精密量取混合对照品溶液适量,用甲醇逐级稀释,制成质量浓度约为75、50、25、10 μg/mL的系列混合对照品溶液。

供试品溶液:取各批酸枣仁,置于烘箱中在50 ℃条件下低温烘干,剥离种皮、种仁,研磨成粉,过40目筛,即得。精密称定种皮、种仁粉末约1 g,置于100 mL具塞磨口锥形瓶中,加入70%乙醇30 mL,密塞,称重质量。超声提取30 min (功率600 W,频率40 kHz)后静置,放冷,再称定质量,用70%乙醇补足减失的质量,摇匀,静置,取1 mL上清液用0.22 μm微孔有机滤膜过滤,待测。取各酸枣仁供试品溶液200 μL,混匀,作为质量控制样品(quality control, QC),在UPLC-Q-TOF/MS和UPLC-CAD分析过程中用于确定结果的重复性。

### 1.3 UPLC-Q-TOF/MS定性分析方法

色谱条件 Waters ACQUITY UPLC HSS T3色谱柱(100 mm×2.1 mm, 1.8 μm);流动相:0.1%甲酸水溶液(A)-乙腈(B);流速:0.5 mL/min;梯度洗脱:0~10 min, 5%B~27%B; 10~12 min, 27%B~41%B; 12~13 min, 41%B~55%B; 13~22 min, 55%B~70%B; 22~25 min, 70%B~95%B; 25~27 min, 95%B; 27~28 min, 95%B~5%B; 28~30 min, 5%B。柱温:40 ℃,进样量:10 μL。

质谱条件 电喷雾离子源(ESI),离子源温度为110 ℃,脱溶剂气体为氮气,脱溶剂气温度450 ℃,脱溶剂气体流量850 L/h,锥孔气体流量50 L/h,采集模式为多能级数据采集(MS^E^)。选择正、负离子扫描方式,扫描范围*m/z* 50~1500,正离子模式下毛细管电压为3 kV,低能量扫描时能量为6 eV,高能量扫描时能量为20~40 eV;负离子模式下毛细管电压为2.5 kV,低能量扫描时能量为6 eV,高能量扫描时能量为50~70 eV。准确质量数用Leucine-enkephalin作校正液。

### 1.4 多元统计学分析

利用UNIFI 1.9.4对所有酸枣仁种皮、种仁样品采集的MS^E^原始数据进行峰提取、对齐、滤噪、归一化等预处理,主要参数设置如下:保留时间范围0.5~26 min,偏差为1.0×10^-5^(10 ppm),保留时间漂移值为0.1 min,得到.USP格式标有峰面积和保留时间的数据表。使用SIMCA 14.0软件对数据进行标准化处理,随后进行PCA无监督模式识别和OPLS-DA有监督模式分析。

### 1.5 UPLC-CAD半定量分析方法

Waters ACQUITY UPLC BEH Shield RP 18色谱柱(100 mm×2. 1 mm, 1.7 μm);分析泵流动相:0.1%甲酸水溶液(A)-乙腈(B)。梯度洗脱条件(分析泵): 0~2 min, 5%B~12%B; 2~4 min, 12%B~16%B; 4~8 min, 16%B~20%B; 8~12 min, 20%B~24%B; 12~13 min, 24%B~36%B; 13~16 min, 36%B~45%B; 16~17 min, 45%B~65%B; 17~24 min, 65%B~70%B; 24~26 min, 70%B~95%B; 26~28 min, 95%B; 28~29 min, 95%B~5%B; 29~30 min, 5%B。流速:0. 5 mL/min;柱温:40 ℃;进样体积:3 μL。补偿泵流动相:0. 1%甲酸水溶液(C)-乙腈(D);启用Chromeleon 7工作站中的“反梯度”功能,设定延迟时间为0.608 min,流速0.5 mL/min,雾化器温度50 ℃,CAD采集频率10 Hz,滤波1 s,幂指数(power function value, PFV)为1.29。使用Chromeleon 7软件(美国Thermo Fisher公司)进行数据采集和分析。

## 2 结果与讨论

### 2.1 成分鉴定

采用UPLC-Q-TOF/MS^E^对酸枣仁种皮、种仁的化学成分进行定性分析,不同部位在负、正离子模式下的基峰强度(BPI)色谱图见图1。结合UNIFI 1.9.4数据管理软件,查阅文献,自建酸枣仁数据库,其中包括化合物名称、分子式、化学结构式和碎片离子信息,根据TOF/MS所得到的精确分子质量信息初步鉴定;再通过对照品、裂解碎片对照、MassBank、PubChem、LIPID MAPS质谱库检索及参考相关文献等方法人工确证,鉴定化合物误差在5 ppm的质量偏差范围内。

**图 1:**
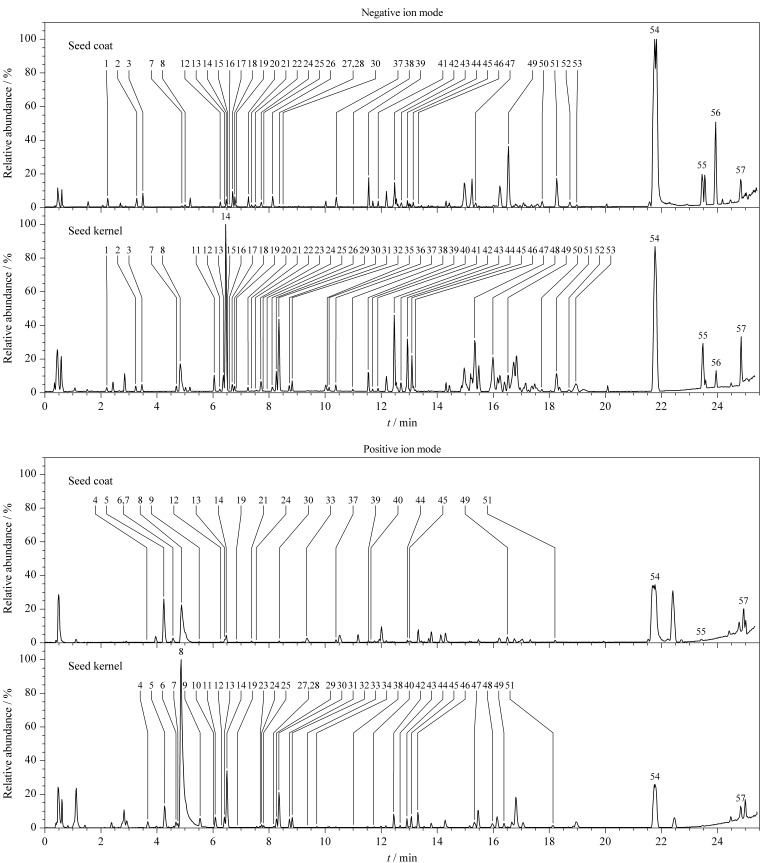
UPLC-Q-TOF/MS^E^分析酸枣仁种皮、种仁部位在负、正离子模式下的BPI色谱图

最终从酸枣仁种皮、种仁中共鉴定出57个化学成分(见表1),包括三萜14个、黄酮23个、生物碱7个、羧酸6个、其他类型成分7个。以酸枣仁皂苷A为例说明鉴定过程,在ESI^-^模式下,峰42在*m/z* 1251.6025处产生[M+HCOO]^-^加合离子,准分子离子峰为*m/z* 1205.5968 [M-H]^-^;在ESI^+^模式下,峰42在*m/z* 1207.6158处产生准分子离子峰[M+H]^+^,推测分子式为C_58_H_94_O_26_。在负离子高能级质谱下产生*m/z* 1073.5538 [M-H-Rha]^-^、 *m/z* 911.5007 [M-H-Rha-Glc]^-^、 *m/z* 749.4480[M-H-Rha-2Glc]^-^、 *m/z* 603.3908[M-H-Rha-2Glc-Xyl]^-^的脱糖碎片离子以及*m/z* 471.3503[M-H-Rha-2Glc-Xyl-Ara]^-^苷元碎片离子;在正离子高能级质谱下产生*m/z* 455.3388和*m/z* 437.3282特征母核碎片离子,结合文献[4]及对照品,鉴定化合物42为酸枣仁皂苷A。

**表 1:** UPLC-Q-TOF/MS^E^鉴定得到的酸枣仁种皮、种仁的化学成分

No.		*t*_R_ in Fig. 1/min		Formula		Precursor ion	Mass		Theoretical mass		Error/ 10^-6^		Fragment ions (*m/z*)			Compound	Seed coat		Seed kernel
1		2.25		C_16_H_18_O_9_		[M-H]^-^	353.0867		353.0873		-1.7		335.0876, 191.0549, 179.0344, 161.0211, 135.0441			neochlorogenic acid^[19]^	+		+
2		3.30		C_16_H_18_O_9_		[M-H]^-^	353.0870		353.0873		-0.9		191.0550, 179.0347, 161.0232, 85.0329			chlorogenic acid^[19]^	+		+
3		3.52		C_16_H_18_O_9_		[M-H]^-^	353.0869		353.0873		-1.1		191.0557, 179.0339, 173.0450, 161.0248, 155.0330, 135.0444,			cryptochlorogenic acid^[19]^	+		+
													93.0338						
4		3.67		C_19_H_24_NO_3_		[M]^+^	314.1760		314.1756		1.3		314.1755, 269.1203, 237.0095, 211.1090, 175.0743, 145.0653,			magnocurarine isomer^[20]^	+		+
													116.9765, 107.0503						
5		4.26		C_17_H_19_NO_3_		[M+H]^+^	286.1447		286.1443		1.4		286.1440, 269.1181, 237.0911, 209.0965, 191.0856, 175.0756,			aconitine^[3]^	+		+
													143.0495, 107.0496						
6		4.66		C_20_H_26_NO_4_		[M]^+^	344.1860		344.1862		-0.6		299.1278, 267.0997, 239.1145, 237.0904, 207.9867, 175.0752,			tembetarin^[19,21]a,c ^	+		+
													137.0577						
7		4.73		C_27_H_30_O_15_		[M-H]^-^	593.1514		593.1506		1.4		473.1082, 395.0771, 383.0767, 353.0657, 325.0708, 297.0758			vicenin-Ⅱ^[4,22]^	+		+
8		4.84		C_20_H_24_NO_4_		[M]^+^	342.1703		342.1705		-0.6		297.1129, 282.0894, 265.0867, 237.0914, 219.0803, 58.0668			magnoflorine^b^	+		+
9		5.51		C_19_H_24_NO_3_		[M]^+^	314.1761		314.1756		1.6		314.1755, 269.1185, 237.0928, 209.0956, 175.0746, 143.0493			magnocurarine^[20,23]^	+		+
10		6.00		C_42_H_48_O_23_		[M+H]^+^	921.2681		921.2665		1.7		759.2183, 741.1762, 639.1682, 447.1330, 429.1185, 411.1058,			6‴-(4‴-*O*-glc)-vanilloylspinosin^[4]^	-		+
													381.0920, 351.0924, 327.0807, 151.0363						
11		6.08		C_27_H_30_O_15_		[M+H]^+^	595.1649		595.1663		-2.4		433.1125, 415.1020, 397.0916, 379.0811, 367.0818, 337.0724,			meloside A^[4]^	-		+
													313.0710, 283.0609						
12		6.29		C_32_H_38_O_19_		[M+H]^+^	727.2081		727.2086		-0.7		595.1814, 581.1440, 449.1064, 287.0556			camelliaside B^[4,23]^	+		+
13		6.40		C_28_H_32_O_15_		[M+H]^+^	609.1804		609.1819		-2.5		447.1285, 429.1191, 411.1051, 393.0976, 351.0873, 327.0871,			isospinosin^[3]^	+		+
													297.0765, 285.0767						
14		6.49		C_28_H_32_O_15_		[M+H]^+^	609.1813		609.1819		-1.0		489.1479, 447.1290, 429.1180, 411.1070, 393.1081, 381.0967,			spinosin^b^	+		+
													351.0864, 327.0868, 297.0757						
15		6.56		C_21_H_20_O_10_		[M-H]^-^	431.0972		431.0978		-1.4		323.0573, 311.0557, 307.0622, 293.0428, 283.0594, 281.0456,			isovitexin^[24]^	+		+
													269.0444						
16		6.67		C_27_H_30_O_15_		[M-H]^-^	593.1511		593.1506		0.8		593.1526, 285.0394			kaempferol-3-*O*-rutoside isomer^[3,25]^	+		+
17		6.73		C_29_H_36_O_15_		[M-H]^-^	623.1985		623.1976		1.4		461.1664, 179.0341, 161.0236			acteoside^[26]a,c ^	+		+
18		6.81		C_29_H_36_O_15_		[M-H]^-^	623.1982		623.1976		1.0		461.0904, 179.0342, 161.0236			isoacteoside^[26]^	+		+
19		6.87		C_22_H_22_O_10_		[M+H]^+^	447.1292		447.1291		0.2		429.1295, 411.1142, 327.0872, 297.0773, 174.0549			swertisin^[27]^	+		+
20		7.29		C_25_H_24_O_12_		[M-H]^-^	515.1195		515.1190		1.0		353.0880, 191.0553, 179.0340, 173.0448, 135.0443			isochlorogenic acid B^[28]^	+		+
21		7.40		C_27_H_30_O_15_		[M-H]^-^	593.1508		593.1506		0.3		285.0388, 284.0316, 255.0291			kaempferol-3-*O*-rutoside^[3,25]^	-		+
22		7.53		C_25_H_24_O_12_		[M-H]^-^	515.1190		515.1190		0		353.0858, 191.0552, 179.0340, 173.0458, 135.0439			isochlorogenic acid A^[28]^	+		+
23		7.70		C_44_H_49_O_22_N		[M+H]^+^	944.2816		944.2824		-0.9		944.2839, 824.2661, 447.1273, 429.1159, 327.0866, 297.1133			6‴-*O*-(3-Glc-indole-acetyl)spinosin or isomer^[4,11]^	-		+
24		7.75		C_37_H_38_O_18_		[M+H]^+^	771.2140		771.2136		0.5		651.1650, 609.1619, 433.1139, 415.1023, 379.0705, 337.0701,			isovitexin-2″-*O*-(6-feruloyl)-glucopyranoside^[4,27]^	+		+
													313.0726, 177.0555						
25		7.82		C_44_H_49_O_22_N		[M+H]^+^	944.2815		944.2824		0.1		944.2797, 824.2675, 447.1271, 429.1161, 411.1082, 327.0849,			6‴-*O*-(3-Glc-indole-acetyl)spinosin or isomer^[4,11]^	-		+
													297.0788						
26		8.15		C_25_H_24_O_12_		[M-H]^-^	515.1185		515.1190		-1.0		353.0862, 191.0551, 179.0338, 173.0447, 135.0443			isochlorogenic acid C^[28]^	+		+
27		8.18		C_39_H_42_O_19_		[M+H]^+^	815.2397		815.2399		-0.3		695.2109, 609.1945, 447.1345, 429.1194, 411.1042, 393.0956,			6‴-sinapoylspinosin^[3,27]^	-		+
													327.0868, 297.0854, 207.0659, 163.0387						
28		8.20		C_43_H_52_O_19_		[M+H]^+^	873.3188		873.3181		0.8		855.3101, 735.2551, 447.1412, 429.1191, 411.1068, 393.0933,			6‴-dihydrophaseoylspinosin^[3]^	-		+
													351.0852, 327.0861, 247.1341, 207.0649, 163.0397						
29		8.30		C_37_H_38_O_17_		[M+H]^+^	755.2174		755.2187		-1.7		635.1722, 609.1754, 447.1285, 429.1180, 351.0857, 327.0863,			6‴-*p*-coumaloylspinosin^[3]^	-		+
													309.0979, 147.0442						
30		8.39		C_38_H_40_O_18_		[M+H]^+^	785.2289		785.2293		-0.5		665.1870, 609.1771, 447.1184, 429.1184, 411.1092, 393.0977,			6‴-feruloylspinosin^b^	+		+
													381.0981, 351.0871, 327.0869, 207.0765, 177.0551						
31		8.74		C_44_H_49_O_22_N		[M+H]^+^	944.2810		944.2824		-1.5		944.2797, 782.2302, 764.2184, 489.1389, 393.0977, 327.0861			3‴-(*N*-*β*-D-Glucopyranosyl)-2‴,3‴-dihydro-2‴-	-		+
																oxo-indol-3‴-yl-acetate spinosin or isomer^[29]^			
32		8.84		C_44_H_49_O_22_N		[M+H]^+^	944.2811		944.2824		-1.4		944.2799, 782.2269, 764.2170, 602.1631, 489.1395, 393.0974,			6‴-(*N*-*β*-D-glucopyranosyl)-2‴,3‴-dihydro-2‴-	-		+
													327.0861			oxo-indol-3‴-yl-acetate spinosin or isomer^[29]^			
33		9.37		C_18_H_19_NO_2_		[M+H]^+^	282.1490		282.1494		-1.4		265.1223, 250.0998, 235.0762, 219.0824, 191.0863			*N*-nornuciferine^[4]^	+		+
34		9.71		C_38_H_40_O_18_		[M+H]^+^	785.2310		785.2293		2.2		665.1846, 447.1279, 429.1184, 411.1117, 327.0863, 177.0545			6‴-feruloylspinosin isomer^[3,4,27]^	-		+
35		10.11		C_54_H_58_NO_25_		[M+HCOO]^-^	1164.3208		1164.3196		1.0		1118.3135, 942.2715, 762.2023, 663.1707, 293.0443			6‴-*O*-(3-Glc-indole-acetyl)-6‴-feruloylspinosin or	-		+
																isomer^[4]^			
36		10.18		C_54_H_58_NO_25_		[M+HCOO]^-^	1164.3210		1164.3196		1.2		1118.3145, 942.2654, 783.2103, 762.2061, 663.1730, 293.0453			6‴-*O*-(3-Glc-indole-acetyl)-6‴-feruloylspinosin^[4]^	-		+
37		10.42		C_48_H_50_O_21_		[M-H]^-^	961.2802		961.2766		3.75		943.2595, 931.2581, 307.0561			6‴-*p*-coumaroyl-6‴-sinapoyl spinosin^[11,30]^	+		+
38		11.01		C_58_H_96_O_27_		[M+HCOO]^-^	1269.6135		1269.6115		1.6		1223.6045, 919.4577, 787.4113, 625.3592, 479.3006, 255.2336			protojujuboside B^[4,11]^	+		+
39		11.56		C_53_H_86_O_22_		[M-H]^-^	1073.5532		1073.5537		-0.5		911.5039, 749.4492, 603.3906, 471.3479, 279.2314			jujuboside Ⅰ^[4]^	+		+
40		11.63		C_31_H_42_N_4_O_4_		[M+H]^+^	535.3268		535.3284		-3.0		148.1116			sanjoinine A^[3]^	+		+
41		11.90		C_53_H_86_O_22_		[M+HCOO]^-^	1119.5603		1119.5587		1.4		1073.5677, 749.4483, 603.3909, 471.3471, 279.2328			jujuboside Ⅰ isomer^[4]^	+		+
42		12.48		C_58_H_94_O_26_		[M+HCOO]^-^	1251.6025		1251.6010		1.1		1073.5540, 911.5007, 749.4480, 603.3908, 471.3503			jujuboside A^b^	+		+
43		12.71		C_58_H_94_O_26_		[M+HCOO]^-^	1251.6018		1251.6010		0.6		1073.5531, 911.4987, 749.4526, 279.2318			jujuboside A isomer^[3,12]^	+		+
44		12.94		C_52_H_84_O_21_		[M+HCOO]^-^	1089.5496		1089.5482		1.3		1043.5425, 911.5007, 749.4480, 603.3908, 471.3448			jujuboside B^b^	+		+
45		13.10		C_55_H_86_O_24_		[M-H]^-^	1129.5452		1129.5431		1.9		1085.5536, 1043.5443, 911.5009, 749.4488, 603.3905,			malonyl-jujuboside B^a,c^	+		+
													471.3534						
46		13.36		C_54_H_86_O_22_		[M+HCOO]^-^	1131.5596		1131.5587		0.8		1043.5497, 911.5013, 749.4485, 603.3876, 471.1332			acetyl-jujuboside B^[4,11]^	+		+
47		15.33		C_27_H_49_O_12_P		[M-H]^-^	595.2886		595.2883		0.5		595.2848, 279.2317, 241.0104			1-(9*Z*,12*Z*-octadecadienoyl)-glycero-3-phospho-	+		+
																(1'-myo-inositol)^a,c^			
48		15.98		C_43_H_79_O_13_P		[M-H]^-^	833.5173		833.5180		-0.8		571.2925, 371.0077, 281.2468, 255.2321, 241.0103, 152.9950,			1-hexadecanoyl-2-(9*Z*,12*Z*-octadecadienoyl)-	-		+
													96.9687			glycero-3-phospho-(1'-myo-inositol) or isomer^a,c^			
49		16.53		C_30_H_48_O_4_		[M-H]^-^	471.3479		471.3474		1.1		471.3474, 279.2320, 183.0129			alphitolic acid^[4,31]^	+		+
50		17.74		C_30_H_46_O_4_		[M-H]^-^	469.3318		469.3318		0		451.3236, 391.2265, 279.2319			zizyberanalic acid^[32]^	+		+
51		18.26		C_30_H_46_O_5_		[M-H]^-^	485.3273		485.3267		1.2		439.3222, 423.3263			ceanothic acid^[31]^	+		+
52		18.73		C_30_H_46_O_4_		[M-H]^-^	469.3320		469.3318		0.4		391.2245, 279.2321			zizyberanalic acid isomer^[32]^	+		+
53		18.94		C_27_H_53_O_12_P		[M-H]^-^	599.3203		599.3196		1.2		315.0501, 283.2631, 255.2322, 241.0107, 223.0011, 152.9950,			1-octadecanoyl-sn-glycero-3-phospho-(1'-myo-	-		+
													78.9684			inositol)^a,c^			
54		21.70		C_30_H_48_O_3_		[M-H]^-^	455.3525		455.3525		0		455.3525, 407.3291			betulinic acid^b^	+		+
55		23.42		C_18_H_32_O_2_		[M-H]^-^	279.2324		279.2324		0		279.2322			linoleic acid^[4]^	+		+
56		23.90		C_30_H_46_O3		[M-H]^-^	453.3370		453.3369		0.2		325.1949, 293.1786, 279.2320, 255.2321			betulonic acid^[4]^	+		+
57		24.81		C_18_H_34_O_2_		[M-H]^-^	281.2476		281.2481		-1.8		281.2487			oleic acid^[4]^	+		+

a. First identified in Ziziphi Spinosae Semen; b. identified by standards; c. identified by online database (e.g., MassBank). +: identified compounds exist in the part. -: identified compounds do not exist in the part.

### 2.2 不同部位酸枣仁多元统计分析结果

为了解酸枣仁不同部位间的总体差异和相同部位的组内差异大小,对酸枣仁种皮、种仁采集的UPLC-Q-TOF/MS^E^数据进行分析。首先利用SIMCA14.0软件进行无监督PCA分析,结果如图2所示,QC样品聚类且居中,证明仪器稳定,分析方法可靠。不同批次酸枣仁的种皮、种仁样品分别聚集,分类趋势明显。

**图 2:**
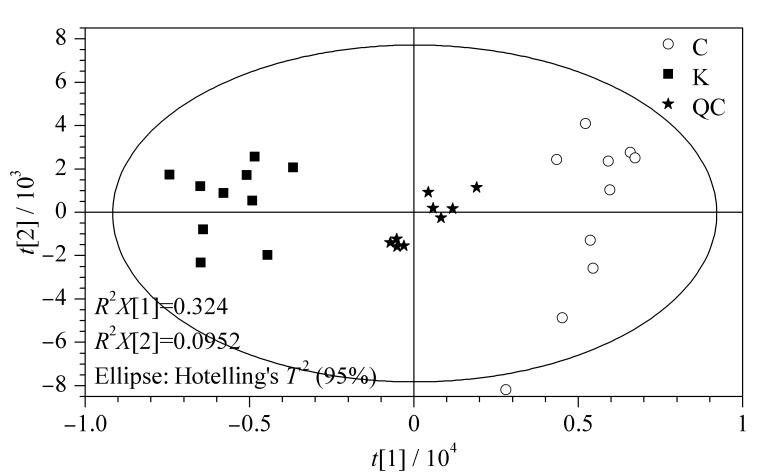
负离子模式下种皮、种仁的PCA得分图

为进一步探究不同部位间的差异成分,采用有监督的OPLS-DA分析。以10批酸枣仁种皮、种仁化学成分的相对峰面积为变量进行分析,结果见图3,种皮、种仁两组差异明显,与PCA结果一致。建立的OPLS-DA模型参数解释率*R*^2^*Y* (cum)为0.991,预测率*Q*^2^ (cum)为0.960,两者接近1,说明该模型能很好地解释和预测种皮和种仁之间的差异,模型预测能力较好,可用于进一步鉴定差异成分。

**图 3:**
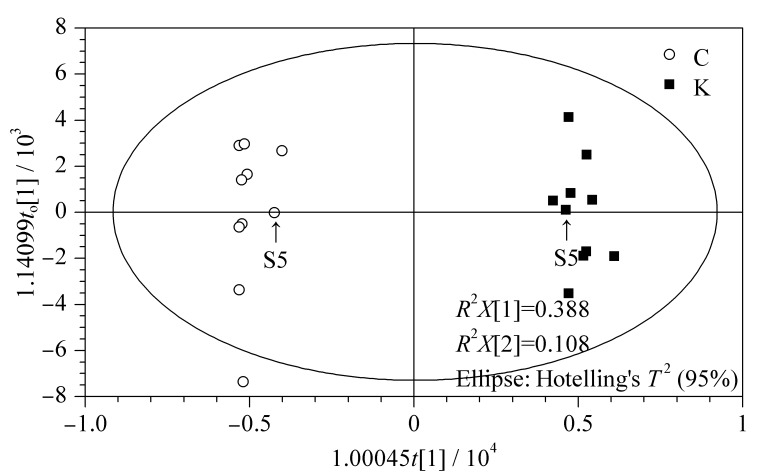
负离子模式下种皮、种仁的OPLS-DA得分图

根据OPLS-DA结果,得S形图(S-plot),可将变量清晰地展示在二维平面图上,位于“S”两翼的变量是两组间差异最为显著的化合物^[33]^,如图4所示,以变量投影重要度(VIP)值>5为筛选标准,共鉴定出17个差异成分,如表2所示。白桦脂酸、麦珠子酸(alphitolic acid)和酸枣仁皂苷Ⅰ主要存在于种皮部位,桦木酮酸(betulonic acid)只存在于种皮部位。斯皮诺素、酸枣仁皂苷A和6‴-阿魏酰斯皮诺素等13个化合物主要存在于种仁部位。

**图 4:**
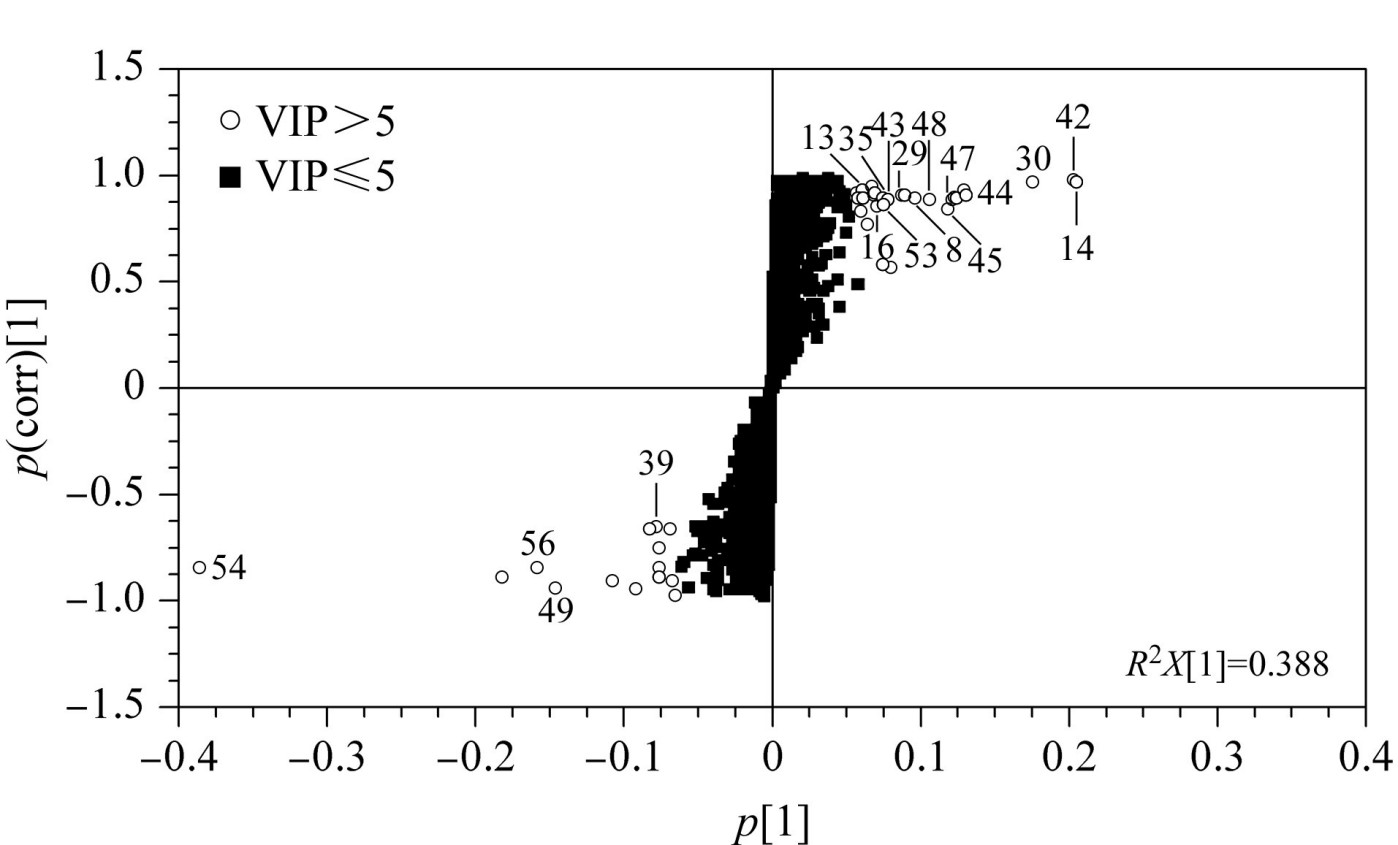
种皮、种仁的S-plot图

**表 2:** 酸枣仁种皮、种仁的差异成分

No.	Peak No.^1)^	t_R_/min	Formula	Compound^2)^	VIP	Main presence part
1	54	21.70	C_30_H_48_O_3_	betulinic acid	31.85	seed coat
2	14	6.49	C_28_H_32_O_15_	spinosin	16.92	seed kernel
3	42	12.48	C_58_H_94_O_26_	jujuboside A	16.85	seed kernel
4	30	8.39	C_38_H_40_O_18_	6‴-feruloylspinosin	14.55	seed kernel
5	56	23.90	C_30_H_46_O_3_	betulonic acid	13.06	seed coat
6	49	16.53	C_30_H_48_O_4_	alphitolic acid	12.05	seed coat
7	44	12.94	C_52_H_84_O_21_	jujuboside B	10.75	seed kernel
8	47	15.33	C_27_H_49_O_12_P	1-(9Z,12Z-octadecadienoyl)-glycero-3-phospho-(1'-myo-inositol)	10.07	seed kernel
9	45	13.10	C_55_H_86_O_24_	malonyl-jujuboside B	9.77	seed kernel
10	48	15.98	C_43_H_79_O_13_P	1-hexadecanoyl-2-(9Z,12Z-octadecadienoyl)-glycero-3-phospho-	8.81	seed kernel
				(1'-myo-inositol) or isomer		
11	8	4.84	C_20_H_24_NO_4_	magnoflorine	7.99	seed kernel
12	29	8.30	C_37_H_38_O_17_	6‴-p-coumaloylspinosin	7.18	seed kernel
13	39	11.56	C_53_H_86_O_22_	jujuboside Ⅰ	6.49	seed coat
14	35	10.11	C_54_H_58_NO_25_	6‴-O-(3-Glc-indole-acetyl)-6‴-feruloylspinosin	6.22	seed kernel
15	11	6.08	C_27_H_30_O_15_	meloside A	5.78	seed kernel
16	32	8.84	C_44_H_49_O_22_N	6‴-(N-β-D-glucopyranosyl)-2‴,3‴-dihydro-2‴-oxo-indol-3‴-yl-	5.66	seed kernel
				acetate spinosin or isomer		
17	13	6.40	C_28_H_32_O_15_	isospinosin	5.07	seed kernel

1) Peak numbers were consistent with Table 1. 2) sort by VIP value.

## 2.3 UPLC-CAD半定量分析结果

### 2.3.1 CAD响应一致性考察

CAD作为一种质量型通用检测器,色谱峰的响应不仅与化合物结构有关,还与进入检测器时流动相的有机溶剂组成有关。中药成分复杂,极性跨度大,其色谱指纹图谱通常采用梯度洗脱。为了使指纹图谱中各个色谱峰的响应尽可能一致,各个色谱峰的面积可以较好地直接反映化合物的量,本工作采用了仪器的流动相反梯度补偿功能,使各分析物具有相同的雾化效率和相近的响应因子,从而建立了UPLC-CAD半定量指纹图谱,以对酸枣仁种皮、种仁中的化学成分进行分析,了解主要成分及其各成分间的含量关系。

首先选取酸枣仁中不同结构类型的6个代表成分:木兰花碱(生物碱)、斯皮诺素(黄酮)、6‴-阿魏酰斯皮诺素(黄酮)、酸枣仁皂苷A(三萜皂苷)、酸枣仁皂苷B(三萜皂苷)和白桦脂酸(三萜酸)。利用对照品测定它们在反梯度补偿前、后在不同浓度下的响应因子(*f*)及其平均响应因子,比较考察不同结构类型化合物的CAD响应一致性,进而评价UPLC-CAD作为半定量指纹图谱的可行性。6个化合物的响应因子采用*f=A_i_/C_i_*计算。其中,*A*为峰面积,*C*为成分的质量浓度。100 μg/mL混合对照品反梯度补偿前、后的CAD图谱如图5所示,补偿前各成分响应因子的RSD值为61.50%,补偿后成分各响应因子的RSD值为7.87%,表明流动相反梯度补偿后6个化合物的响应一致性好。混合对照品在不同浓度下的反梯度补偿后的响应因子测定结果见表3,在100、75、50、25和10 μg/mL下,6个化合物的响应因子间的RSD值分别为7.87%、9.43%、5.41%、6.53%和9.37%,同时计算了各浓度下6个化合物平均响应因子间的RSD值为7.04%(小于10%),说明各化合物响应趋于一致。因此,建立的UPLC-CAD结合反梯度补偿半定量指纹图谱使各成分响应一致性良好,不仅可以利用峰面积反映不同部位样品之间各个成分含量的差异,而且可以直观地了解同一样品中主要化学成分及其各个成分间的含量关系。

**图 5:**
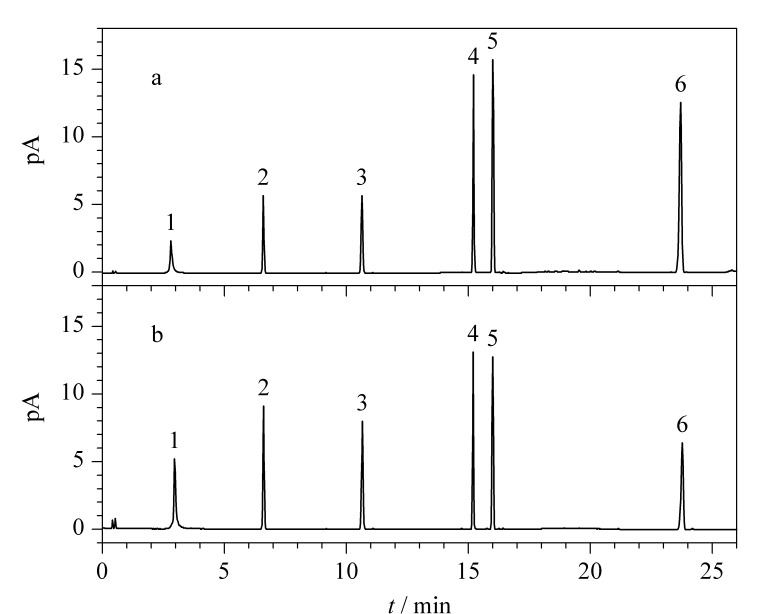
100 μg/mL混合对照品溶液在流动相反梯度补偿(a)前、(b)后的UPLC-CAD图谱

**表 3:** 不同浓度下各个对照品反梯度补偿后的响应因子

Mass concentration/(μg/mL)	f_Magnoflorine_	f_Spinosin_	f_6‴-Feruloylspinosin_	f_Jujuboside A_	f_Jujuboside B_	f_Betulinic acid_	RSD/%
100	0.005242	0.005262	0.005430	0.005395	0.005767	0.006391	7.87
75	0.004873	0.005108	0.005215	0.005203	0.00556	0.006309	9.43
50	0.005095	0.005231	0.005323	0.005534	0.005833	0.005757	5.41
25	0.004915	0.005239	0.005250	0.005268	0.005787	0.005812	6.53
10	0.004299	0.005059	0.005099	0.005519	0.005632	0.00542	9.37
Mean	0.004885	0.005180	0.005264	0.005384	0.005716	0.005938	7.04

### 2.3.2 样品半定量分析

对不同酸枣仁样品的种皮、种仁进行半定量分析,每个样品平行取样3次。以S5样品的种皮、种仁部位的CAD半定量指纹图谱为例,阐述不同部位的主要化学成分,如图6所示。除色谱柱不保留的酸枣仁寡糖等成分外,种皮中表征的主要化学成分为白桦脂酸和油酸,其中白桦脂酸约占种皮总成分含量的22.59%,油酸约占2.99%;种仁部位中表征的主要成分为斯皮诺素、酸枣仁皂苷A、亚油酸、白桦脂酸和油酸,斯皮诺素、酸枣仁皂苷A和白桦脂酸分别约占种仁总成分的1.33%、1.01%和1.62%,亚油酸和油酸两者共占种仁总成分含量的5.20%。

**图 6:**
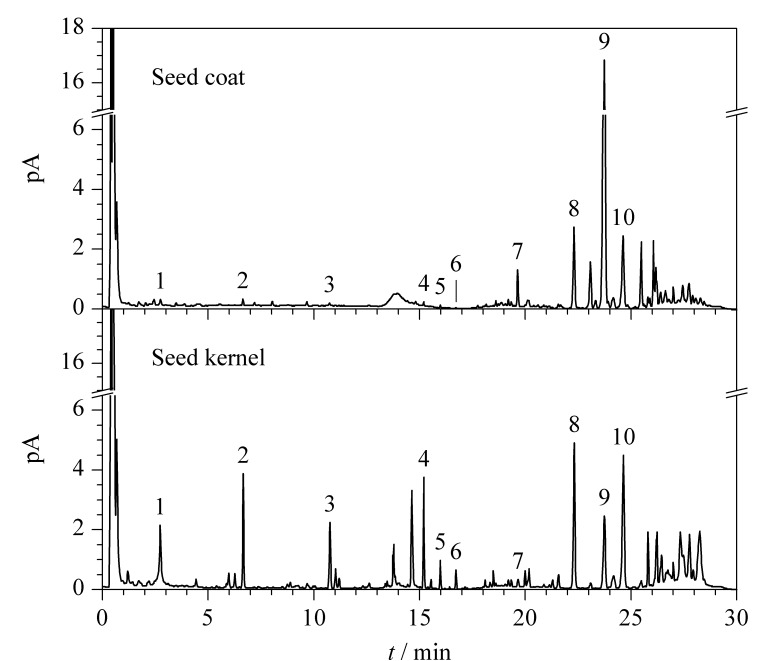
样品S5种皮、种仁的UPLC-CAD半定量指纹图谱

使用Origin 14软件绘制柱状图,以成分为横坐标,以平均峰面积为纵坐标,比较10批酸枣仁种皮、种仁之间成分的含量差异,如图7所示。种皮部位中白桦脂酸和麦珠子酸的相对含量高于种仁,其中白桦脂酸的相对含量是种仁的7倍;其余8个成分均在种仁中含量更高,如种仁中酸枣仁皂苷A、斯皮诺素、木兰花碱和油酸的相对含量分别是种皮的24倍、18倍、7倍和2倍。

**图 7:**
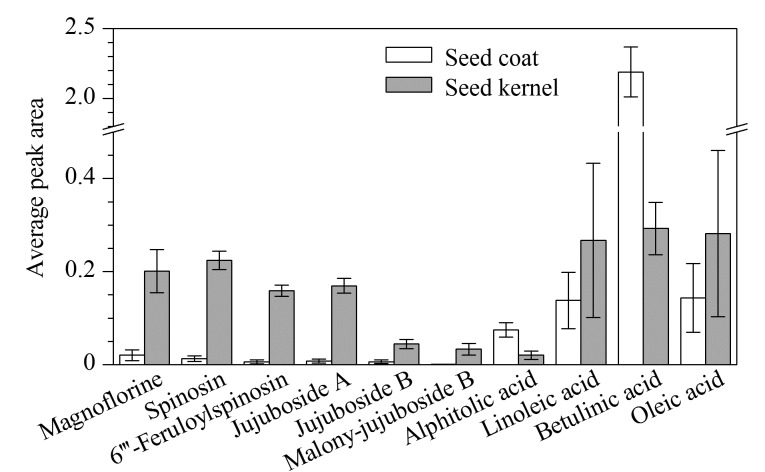
酸枣仁样品种皮、种仁中10个主要成分的相对含量(*n*=10)

中药成分组成复杂,一个或几个成分的含量往往难以反映中药的质量。目前液相色谱-质谱联用技术的发展,已经可以较好地解决中药中成分的定性问题,可以表征成分的组成,明确各个峰的具体结构。多成分的含量测定也是解决中药质控的一种手段,当定量测定的化合物较多时,工作量巨大,有时还受到能否获得对照品的制约。液相色谱指纹图谱是目前进行中药整体质量控制的主要方法,通常使用的紫外类检测器及质谱类检测器峰响应与化合物的具体结构有关,指纹图谱中各个峰的响应不能反映其在药材中的含量情况。CAD作为一种质量型通用检测器,对不同结构类型的化合物均有响应,其响应大小取决于化合物的量,正好可以解决液相色谱指纹图谱中各个色谱峰相对含量的问题,以了解中药中的主要成分以及各个成分间的含量关系。该实验通过酸枣仁中6个不同结构类型化合物响应一致性考察,验证了结合反梯度补偿建立的UPLC-CAD指纹图谱中各个色谱峰的峰面积与含量之间的一致性。相较于含量测定方法,该半定量分析方法不仅能整体反映酸枣仁不同部位中成分的组成,明确各自的主要成分以及各个成分间的含量关系,同时可以考察10个甚至更多成分的相对含量,工作量远小于多个成分的含量测定,更具可行性。

## 3 结论

本研究建立了对酸枣仁不同部位化学成分定性及半定量的分析方法,基于UPLC-Q-TOF/MS对酸枣仁种皮、种仁的化学成分进行了定性分析,明确了种皮、种仁的化学成分组成。通过多元统计分析,筛选出两者间17个差异成分,了解了酸枣仁两个部位的成分差异,为后续酸枣仁不同部位化学成分的鉴定及质量研究提供参考。

同时,本研究建立了酸枣仁种皮、种仁反梯度补偿下的UPLC-CAD指纹图谱,直接利用各峰峰面积对酸枣仁不同部位的主要化学成分进行半定量分析,阐明了各化合物在同一部位及不同部位之间含量的关系。目前研究显示酸枣仁中发挥镇静催眠作用的主要成分为斯皮诺素、酸枣仁皂苷A、6‴-阿魏酰斯皮诺素和酸枣仁皂苷B等,它们在种仁中含量高,而种皮中含量低,为失眠患者使用酸枣仁时为保证口感去除种皮提供了科学依据。同时白桦脂酸作为种皮的主要成分,被认为是一种天然抗癌化合物^[34]^,可用于预防、抑制多种肿瘤,因此无论是种皮还是种仁均有良好的活性研究价值,进而有助于后续对酸枣仁不同部位的合理利用与开发。
